# How does Confucian culture affect technological innovation? Evidence from family enterprises in China

**DOI:** 10.1371/journal.pone.0269220

**Published:** 2022-06-08

**Authors:** Yi Chen, Ping Lin, Hai-Tao Tsao, Shaofei Jin

**Affiliations:** 1 Economics and Management College, Minjiang University, Fuzhou, China; 2 Rural Revitalization Research Institute, Minjiang University, Fuzhou, China; 3 Center of Marine Tourism Research, Fujian College’s Research Base of Humanities and Social Science, Minjiang University, Fuzhou, China; Institute for Advanced Sustainability Studies, GERMANY

## Abstract

Culture is one of the crucial elements of technological innovation. The existing studies hold that Confucian culture is conducive to the technological innovation of Chinese Listed Companies. However, Chinese family enterprises with relatively profound Confucianism encounter the bottleneck of weak innovation. This makes people wonder whether Confucian culture is conducive to the technological innovation of family enterprises. To solve this mystery, we investigated the effects of Chinese Confucianism on technological innovation in Chinese family enterprises. We found that family entrepreneur’s entrepreneurship had worse innovation performance under the influence of Confucian culture. The results are robust to different measures of innovation and are still valid when controlling for the potential endogeneity between Confucian culture and technological innovation. This study provides a more fine-grained perspectives about Chinese innovation culture.

## Introduction

In uncertain situation, facing the triple challenges of "inheritance, transformation and entrepreneurship", innovation has become a unique path for the sustainable development of family enterprises in China [1–6]. It is undeniable that innovation largely depends on culture [7–10]. Under nearly identical conditions of opportunity and probability of success of potential invention and creation, it is impossible for one society to generate enough innovation and invention to promote technological progress and economic growth without providing incentives for the accumulation of human capital and material capital. The incentive mechanisms of capital accumulation lie in institutional arrangements and cultural background. That is, only in a proper institutional environment will the main body of economic activities generate strong motivation for human capital and material capital accumulation. Thus, differences in technological innovation “can be explained satisfactorily only when cultural factors are considered” [11–14]. As an emerging economy with an inherent Eastern cultural atmosphere, China has a large number of informal institutions [15]. This poses higher cultural demands on the sustainability of technological innovation in Chinese family enterprises.

### Limitations of existing theoretical research

Prior studies on the impact of Confucian culture on innovation have been limited to theoretical analysis [16, 17]. The dynamic evolution of formal and informal institutional overlap is also an important but underexplored phenomenon [18]. The neglect of informal institutions (for example, culture and habits) and the interaction between formal institutions and Confucian culture will lead to the distortion of analytical results [19–21]. We believe that informal and formal factors need to be examined together in terms of their mutual interplay, not only because taking a holistic approach provides us with a more complete understanding of innovation activities but also because the interrelationship of the two sets of elements has implications for the performance of innovation behavior. Hence, this study is a tentative attempt to fill this gap and propose a path to independent innovation with Chinese characteristics by examining how Confucian culture, as an important aspect of Chinese culture, impacts innovation in contemporary Chinese companies. The discussion also adds to other studies challenging previous approaches that assume a clear separation between formal and informal institutions when investigating the role of innovation [22].

### Extension of existing empirical studies

For empirical tests, we used the survey data of family enterprises in Growth Enterprise Market (GEM) and the Science and Technology Innovation Board to investigate the impact of Confucian culture on the degree of technological innovation from the perspective of employees. Furtherly, in robustness test, we also used the data of family enterprises in Growth Enterprise Market (GEM) and the Science and Technology Innovation Board as research samples to examine the influence of Confucian culture on innovation, thereby offering insights into the driving effect of Confucian culture. The study follows prior studies [23] by calculating and hand-collecting a set of variables via CSMAR data (https://cn.gtadata.com/) and then provides strong evidence to show that Confucian culture is significantly negatively associated with innovation of Chinese family enterprises which is contrary to previous studies about A-share listed Chinese enterprises.

This paper makes three contributions to the existing literature. First, many scholars have studied the deeper layer of institutional factors that affect the economy, such as culture, religion and customs, since Williamson (2000) [24] proposed the new institutional economics (NIE) analytic framework. Recent studies have shown that cultural factors can influence economic behavior in many ways [25–29], but most studies have relied on Western cultural characteristics (e.g., [30]) or national culture, such as individualism and collectivism (e.g., [29]), as direct proxies for culture; thus, these studies failed to uncover the processes underlying the relationship between Eastern cultural characteristics and innovation. Extending previous literature on the association between culture and innovation [30–35], this study further examines the impacts of Confucian culture on Chinese innovation, a specific issue that is related to technological development and business ethics. This paper is the first to empirically investigate how Confucian culture, as an important cultural factor, influences Chinese technological innovation in the Chinese family enterprises.

Second, most existing studies the effect of culture on innovation have focused on A-share listed Chinese enterprises. Few studies have selected family enterprises as the research object via a quantitative methodology [4, 16, 17]. This study fills this gap by exploring the influences of Confucian culture on innovation in Chinese family enterprises by using quantitative secondary data analysis. In doing so, this paper expands the current thinking of the institution of technological innovation and provides empirical support for exploring innovation practices of Chinese family enterprises.

Third, this paper is one of few studies to employ geographic-proximity-based Confucian culture variables to examine the influence of Confucian culture on technological innovation. In line with prior studies on religion and management [36–40], this paper calculates the geographic distance between a firm and the nearest Confucius temples based on the Google Earth digital map and the equation from the geographic information system. This method is based on two fundamental hypotheses: (1) distance has important impacts on corporate behavior by shaping the extent of information asymmetry, and (2) the geographical distance between a firm and Confucius temples can affect the Confucian cultural atmosphere in the region where the firm is located. This method also provides a vital complementarity to previous studies using the questionnaire method and mitigates criticisms of subjective measures [39]. The number of Confucian temples surrounding the company is less likely to have an endogenous relationship with technological innovation. Thus, endogeneity problems are well avoided.

## Research background

Confucian culture was from in China’s Spring and Autumn Period and has since become embedded in Chinese culture and society, although other ideologies have been more popular at different times. The richness of Confucian culture is the result of its adaptation to ancient China’s feudal patriarchal system and unified regimen of autocratic monarchy as well as its status as a legitimate ideology among philosophers [41–43]. China has witnessed different iterations of Confucian culture, from *Dong Zhongshu* “rejecting all kinds of theoretical schools but Confucius,” to the Confucian school of idealist philosophy of the Song and Ming Dynasties, and then to Ming emperor *Zhu Yuanzhang*’s tenet that “education is key to ruling a nation and school is the basis of education.” Confucian culture has had profound effects on Chinese society through its integration with the country’s feudal monarchy.

Nevertheless, attacks by Western naval powers in the aftermath of the First Opium War (1839–1842) forced China to trade with the Western world on Western countries’ terms, exposing China to Western cultures. Chinese people then began to assimilate Western technologies and political ideals. Reflecting on their own cultural traditions, many Chinese came to see Confucianism as an obstacle to reform and modernization in China. This led to the emergence of anti-tradition and anti-Confucianism reform movements. In 1905, the Chinese government abolished the imperial examination selection test, which had been used to select candidates for the civil service since the Han dynasty. Confucianism shaped the purpose and content of the examination, which was intended to achieve a meritocratic Chinese society (itself a Confucian ideal) and tested candidates’ knowledge of Confucian texts. The exam’s abolition heralded the marginalization of Confucian culture in modern China, and Confucian ethics became cultural fragments of mainstream Chinese culture [44].

However, there remain signs of the influence of Confucian culture on Chinese society. In recent years, the government of China promoted the Confucian ideal of rationalism, adopting measures to promote the legacy of Confucian culture. In 1994, the Ministry of Civil Affairs approved the China Confucian Temple Protection Association, which was mainly responsible for the protection, utilization and research of Confucian memorial buildings, such as Confucian temples. In 2004, the Chinese government funded the establishment of the world’s first Confucius Institute in Seoul. By 2016, China had constructed 511 Confucius Institutes and Confucius classes in 1,073 middle and primary schools in 140 countries and regions (*General Office of the CPC Central Committee and General Office of the State Council*, 2017). After the 18th National Congress of the Communist Party of China in November 2012, the Chinese government announced policies to revitalize Confucianism and other elements of traditional Chinese culture. At the opening ceremony of the International Academic Seminar on the 2565th Anniversary of Confucius’ Birthday, General Secretary Xi Jinping commented that “the rich philosophical thoughts, humanistic spirits, social civilizing ideas and moral philosophies in excellent Chinese traditional culture can provide beneficial enlightenments on understanding and reconstruction of the world, strategy of ruling a country and moral construction.”(http://www.xinhuanet.com/politics/2014-09/24/c_1112612018.htm.) In January 2017, the General Office of the CPC Central Committee and General Office of the State Council released *Opinions on the Implementation of the Excellent Chinese Traditional Cultural Inheritance*, guidelines aimed at preserving and developing traditional Chinese culture in China and on the world stage(http://www.gov.cn/zhengce/2017-01/25/content_5163472.htm).

Indeed, Confucian culture contains rich insights into economic and ethical issues. Through its heritage spanning thousands of years, Confucian culture has formed a cultural gene and become the “law of habit” that guides and restricts the economic life of Chinese people. Moreover, Confucian culture has influenced the construction of modern Chinese ethics.

Chinese family enterprises are selected as the research object of "Confucian culture and technological innovation" in view of the following factors. First, the 30 years of reform and opening up are the 30 years of rapid development of China’s private economy. Nowadays, the private economy has occupied the socialist market, in which family enterprises continue to emerge and develop. As a way of life and attitude, Confucian culture is deeply rooted in family enterprises. Family businesses tend to be more frugal and conservative and lack the ability to innovate.

Second, the existing studies hold that Confucian culture is conducive to the technological innovation of Chinese Listed Companies [45, 46]. However, Chinese family enterprises with relatively profound Confucianism encounter the bottleneck of weak innovation. This makes we wonder whether Confucian culture is conducive to the technological innovation of family enterprises. Thus, the main goal of this research is to examine family enterprises in order to compare with the existing conclusions. The next section of this paper explores the roles of Confucian culture, which has shaped the Chinese government’s agenda of socialist economic development with Chinese characteristics.

## Theoretical deduction and hypotheses

### Theoretical deduction

Among the evolving theories of economic growth, two theoretical trends have become increasingly mainstream in the interpretation of development issues. One trend is to attribute economic growth to whether a society can produce enough good inventions, creations and innovations [47–50]. The second is system determinism; that is, the performance of economic growth depends on whether an effective institutional arrangement can be formed to protect property rights so that the inventor can be rewarded [51–54]. There is hardly any debate on a key proposition in the institution-based view of economic behavior, namely, that institutions matter [55–57]. In general, the institutional arrangements that protect property rights and enable creative people to be rewarded determine economic growth performance and even the rise and fall of countries [58–61].

Due to free riding and transaction costs, effective institutional arrangements may not be automatically generated or smoothly implemented. Ideologies can affect free-riding behavior and transaction costs in institutional operations and changes [62–64]. As the foundation of ideology, culture influences transaction costs [65–69] and then affects technological innovation [70–74]. Culture unifies people’s behavior, but it may also create barriers between people [75]. That is, culture can contribute to or hinder the process of developing and implementing new ideas. Thus, innovation faces the consequences of culture for various reasons.

Culture is deemed to be a crucial basis for innovation in various respects. Cultural ideas and social culture have profound effects on people’s thoughts and behavior [76, 77]. Confucian culture plays an important role in China’s economic life by influencing human thinking and behavior. Although many scholars have studied Confucian culture from philosophical, political and ethical perspectives, few studies to date have examined Confucianism from an economic perspective [44]. Gu (2015) [44] reported that the lack of economic analysis of Confucian culture might be related to the traditional Confucian saying of “unexpressive benefits” (Analects of Confucius—*Zihan*). Indeed, sociological studies have shown a considerable influence of culture on economic behaviors [77–79]. In particular, Confucian culture has become an “unconscious” ethics that guides economic behaviors in the Confucian cultural atmosphere of modern Chinese society [80].

### Hypotheses

Culture has profound effects on human behavior and thinking [77]. Since technological innovation is an activity that requires teamwork and the exchange of thoughts, culture may indirectly influence organizations’ efficiency in technological innovation by unconsciously affecting cooperation and communication between innovation team members. In particular, Confucian culture, which has been rooted in China for thousands of years, may influence the success of organizations’ technological innovation efforts.

First, filial piety extends from family to society, with the thoughts of respecting elders and being loyal to monarchs. Confucian culture requires people to be loyal to their faith and dedicated to their careers, which may reduce the negative effects of agents’ cautiousness with regard to innovation. Innovation is a high-risk activity that requires long time frames but lacks comparability and predictability; these factors contribute to the relatively high agency costs of innovation [81–84]. Confucian culture can enhance the mutual trust between clients and agents. Confucian culture emphasizes that the “The gentleman sees righteousness, whereas the petty man sees profit” and advocates “faithfulness and loyalty” as well as “unexpressive benefit”. Confucian culture advises people to regularly engage in self-introspection and to care little about personal gains or losses. Despite intergenerational transitions in Chinese culture, many Chinese clients and agents support the Confucian view of “righteousness and benefits” as well as “faithfulness.” These views can enhance the mutual trust between clients and agents and lower agency costs [44].

Second, the Confucian idea of “benevolence” can promote teamwork and innovation. Scientific research involves complex, arduous teamwork. Cooperation between team members is vital to complete scientific research. The essence of Confucian culture mainly lies in benevolence. Confucian “benevolence” asks team members not to “only support their own parents and care for their own children but to ensure that all elderly are cared for properly and the young developed fully”. This idea emphasizes mutual support among team members to form close cooperative relations and facilitate the smooth accomplishment of innovation tasks. In addition, “free-riding” in team activities can reduce team members’ work enthusiasm. Confucian culture embeds the professional ethics of “faithfulness and loyalty” as well as the value that “the gentlemen see righteousness” [44], which may enhance the quality of individual work output and prevent free-riding among team members.

In summary, Confucian culture can facilitate technological innovation indirectly by lowering agency costs and enhancing cohesiveness in innovation teams. Specifically, Confucian culture emphasizes the values of “righteousness and benefit” and “faithfulness” that can support the mutual trust between clients and agents to reduce agency costs, increase job stability for scientific researchers, and strengthen the cohesiveness of innovation teams [85]. These factors emphasize the need for empirical research to verify whether Confucian culture can facilitate technological innovation [86]. Given that formal institutions (such as laws) are not perfect, informal institutions (such as culture) may play an important role in economic activities [19, 21, 36, 87]. Hence, the first hypothesis is proposed:

*H1*: *Family enterprises with the Confucian values of benevolence and righteousness perform better in technological innovation than other enterprises*.

On the other hand, Confucian culture may restrict enterprise innovation. The patriarchal hierarchy that Confucianism advocates expands the power distance against free and equal expression by subordinate members. This reduces the efficiency of innovation information transmission. Since innovation is born of sociality and teamwork, it is generated through the exchange and collision of opinions [88]. As direct creators of enterprise value, junior staff may understand enterprise problems better than senior managers. This indicates that junior employees’ suggestions might be more beneficial than superiors’ suggestions for enterprise innovation. However, Confucianism advocates strict obedience to the patriarchal hierarchy. The patriarchal hierarchy extends the power distance so that subordinates cannot express their opinions freely and fairly, thus significantly hindering their contribution to innovation. Classical Confucian texts have recorded this dynamic throughout Chinese history. *The Analects of Confucius* said that “children shall admonish parents with respect to the wrong cognition. If parents refuse to take this advice, children shall execute ‘parents’ orders’ without regrets.” This implies that the expansion of the power distance will discourage employees from expressing opinions even if they have the courage to innovate. This can be explained from two perspectives. On the one hand, innovative information that employees provide to enterprise management might be diluted gradually with the expansion of the power distance. On the other hand, enterprise executives might overlook suggestions from junior employees due to status hierarchy bias. In other words, the patriarchal hierarchy of Confucian culture decreases the supply and adoption efficiency of innovative information, thereby inhibiting enterprise innovation.

Furthermore, Confucian culture advocates harmoniousness: “The most valuable use of the rites is to achieve harmony.” This emphasizes the merits of harmonious relations between individuals and groups, which lead to discourage personal independence or the standardization of individual rights by formal institutions [89]. Jin et al. (2017) [89] reported that Confucian culture may diminish enterprises’ willingness to assume risks and that innovation, as an especially risky objective, might be hindered by Confucian culture. Furthermore, the view in Confucian culture that men are superior to women restricts women’s contributions to innovation. Based on the above analysis, the second hypothesis is proposed:

*H2*: *Family enterprises influenced by Confucian culture demonstrate worse performance in technological innovation than other enterprises*.

## Materials and methods

### Modeling and variable definitions

To study the influences of Confucian culture on technological innovation from the perspective of enterprises, this paper constructs model (1) with reference to the existing research findings [90]:

$$Innovation=\beta_{0}+\beta_{1}CONF+\beta_{2}FA+\beta_{3}LEV+\beta_{4}ROA+\beta_{5}TobinQ+\beta_{6}AGE+\beta_{7}RATIO+\varepsilon$$ (1)

where the explained variable *Innovation* is technological innovation, and the explanatory variable *CONF* is the degree of Confucian culture. The definitions and estimations of all variables are listed in Table 1.

**Table 1:** Name and definition of variables used in the analysis.

Variables	Signs	Definition	Data source
Technological innovation	*Innovation1*	Natural logarithm of total application number of invention patents, patents for utility models and appearance design patents +1	CSMAR
	*Innovation2*	Natural logarithm of capitalization of R&D expenditure	
Confucian Culture	*CONF1* *CONF2* *CONF3*	Confucian temples as percent of all temples within a radius of 100, 200, 300 kilometers around the registration place of the listed companies	Author’s Calculation based on CSMAR, Google- earth map, and GIS
Fixed Asset Share	*FA*	Natural logarithm of (Fixed assets at the end of term + depreciation) / Total assets at the end of term	CSMAR
Asset-liability Ratio	*LEV*	(Short-term borrowing + long-term borrowing + non-current liability due in one year) / total assets	CSMAR
Return on Total Assets	*ROA*	Net profits / total assets	CSMAR
*TobinQ*	*TobinQ*	Market value / total assets	CSMAR
Listed Years	*AGE*	Current year–year of listing + 1	CSMAR
Managers’ Ratio	*RATIO*	Proportion of senior management family members (%)	CSMAR

#### Innovation

Technological innovation is measured by the number of patent applications. The following two indicators are used: (1) the natural logarithm of the total number of patent applications, i.e., the number of patent applications for invention, utility model and design plus 1, which is recorded as *Innovation1*; and (2) the natural logarithm of capitalization of R&D expenditure, which represents the output of R&D, is recorded as *Innovation2*.

#### Confucian culture

This paper selects Confucian temples within different geographical radii, including 100 kilometers, 200 kilometers and 300 kilometers, of the registered area around the listed companies to measure the impact of Confucian culture, which is recorded as CONF1, CONF2, CONF3 [26, 39, 91]. We calculate the geographic distance between a firm and the nearest Confucius temples based on Google-earth digital map and the equation from geographic information system. This method is based on two fundamental hypotheses: (1) the distance has important impacts on corporate behavior by the way of shaping the extent of information asymmetry, and (2) The geographic distance between a firm and Confucius temples can affect Confucian culture atmosphere in a region where a firm is located [39]. So this method provides a vital complementarity to previous studies using questionnaire method, and mitigates rebukes derived from subjective measures.

The possible limitations of measurement variables are a common problem that almost all similar studies should face with. It is reasonable to take the filial piety attitude index as the proxy variable of Confucian culture, using a single index to measure is easy to simplify complex economic and social phenomena, and may even lead to some misunderstanding. Similarly, we do not think that the measurement of Confucian culture provided in this paper are enough to reflect the full view of Confucian culture. On the contrary, we recognize and affirm that the connotation other than familism in Confucian culture can not be reflected by the measurement indicators in this study. Thus, this paper also notes that the measurement indicators adopted must be consistent with the theoretical concept as much as possible.

#### Control variables

We incorporate several control variables assessed by firm characteristics. To avoid confounding the hypothesized effects of companies’ characteristics on their innovative behavior with alternative explanations, we control for Fixed Asset Share, Asset-liability Ratio, Return on Total Assets, *Tobi*,*nQ*, Listed Years, Managers’ Ratio [92, 93].

### Data source and sampling

The data of Chinese family companies in Growth Enterprise Market (GEM) and the Science and Technology Innovation Board provided by the *China Stock Market and Accounting Research (CSMAR)* (http://cn.gtadata.com/) database were used in the influence quantification of Confucian culture. There were 1360 observed values of initial samples, which were screened by the following rules: (1) ST and *ST enterprise samples were deleted; (2) Financial listed companies were deleted; and (3) Samples with missing data were deleted. In the end, 756 observed values were retained. To eliminate the influences of extreme values, all the continuous variables were processed and winsorized at the 1% and 99% quantiles. All the data used in this study can be found in the S1 Raw data.

## Results

### Descriptive statistics

The descriptive statistics of relevant variables are shown in Table 2. The mean and variance of *Innovation1* were 2.57 and 1.20, respectively. The control variable data characteristics were basically consistent with previous studies.

**Table 2:** Descriptive statistics.

Variables	Minimum	Maximum	Mean	Std. Deviation
*Innovation1*	0	5.25	2.57	1.20
*CONF100*	0	9	3.29	2.49
*CONF200*	0	11	6.36	2.95
*CONF300*	0	15	9.43	3
*FA*	14.81	23.85	19.21	1.29
*LEV*	0.04	1.69	0.35	0.19
*ROA*	-19.67	0.42	-0.06	1.15
*TobinQ*	0.19	10.17	2.03	1.39
*AGE*	2	12	7.34	2.92
*RATIO*	0	0.75	0.17	0.15

### Regression results

The regression results of the influence of Confucian culture on enterprise innovation are listed in Table 3. The corresponding coefficients of CONF were significantly negative at the 10% level. All the data proved that the degree of technological innovation increased with Confucian culture.

**Table 3:** Coefficients of regression model.

Variables	Model 1	Model 2	Model 3
*CONF100*	-0.087*		
	(-1.86)		
*CONF200*		-0.066*	
		(-1.80)	
*CONF300*			-0.063*
			(-1.81)
*FA*	0.030*	0.034*	0.036*
	(-0.33)	(-0.37)	(-0.39)
*LEV*	0.232	0.18	0.142
	(-0.68)	(-0.53)	(-0.41)
*ROA*	0.066	0.007	0.007
	(-0.19)	(-0.02)	(-0.02)
*TobinQ*	0.078**	0.063*	0.055*
	(-1.86)	(-1.56)	(-1.37)
*AGE*	0.0259	0.009	-0.002
	(-0.68)	(-0.26)	(-0.06)
*RATIO*	0.311	0.199	0.129
	(-1.5)	(-1.04)	(-0.68)
*_Constant*	1.921	2.169	2.420
	(-1.09)	(-1.22)	(-1.34)
*AdjR-squared*	0.108	0.111	0.103

Note

* = p < 0.10

** = p < 0.01

*** = p < 0.001.

### Robustness test: Substantive variables of innovation

The natural logarithm of number of R&D personnel is used as a substantive variable of innovation for the regression analysis. The robustness test are listed in Table 4. The conclusions are basically the same as the above.

**Table 4:** Robustness test: Using substantive variables of innovation.

Variables	Coefficient of model		
*CONF100*	0.028	(-1.82)	*
*control variable*	*control*		
*Adj*.*R*^*2*^	0.084		

Note

* = p < 0.10

** = p < 0.01

*** = p < 0.001. T-statistic is in brackets.

## Discussion and conclusion

Institutional theory suggests that culture affects innovation [29, 69], but most studies have relied on Western cultural characteristics [7, 30] or national culture (e.g., individualism, collectivism) [29] as direct proxies for culture, such that they fail to uncover the underlying processes of the relationship between Eastern cultural characteristics and innovation. Extending prior literature on the relationship between culture and innovation [31, 32], this study further examines the impacts of Confucian culture on the innovation performance of Chinese family firms. Using a sample of Chinese family enterprises in Growth Enterprise Market (GEM) and the Science and Technology Innovation Board in 2019 and a set of geographic proximity-based Confucian culture variables, this study finds that Confucian culture significantly hinders technological innovation for Chinese family enterprises.

In addition to the theoretical contributions documented in Section 1, this study has several important implications for technological development and business culture. *First*, the Confucian philosophical system continues to have a great impact on Chinese contemporary society and thus has an important influence on independent innovation in China. In fact, the associations between Confucian culture and innovation are implicit, but the influence of cultural factors on innovation is durable, continuous, and stable. Therefore, with regard to Chinese innovation, culture, such as Confucianism, should not be ignored. Moreover, our finding lends important support to Williamson (2000) [24], who emphasizes that researchers should notice the crucial role of informal systems (e.g., religion, belief and culture) in shaping formal institutions and innovation efficiency. However, the results of this study suggest that Confucian culture has a negative and significant impact on innovation in the case of Chinese family enterprises in Growth Enterprise Market (GEM) and the Science and Technology Innovation Board, which contrary to the view that national culture promotes innovation, as described in the literature review (e.g., [94]).

*Second*, our results are also different from prior studies from the perspective of Western culture (e.g., [29, 95–102]) and Western studies on Confucian culture (e.g., [103, 104]). Our results show that Confucian culture sticks to stereotypes and lacks innovation in Chinese family enterprises, which echoes the view about the negative relationship between Confucian culture and innovation in Korean case [104]. This result also reveals the great difference between family enterprises and A-share listed companies in China.

### Enlightenments, limitations and avenues for further research

Confucian culture is rooted in Chinese society. With the increasing integration of Chinese and Western cultures, attitudes toward and treatment of traditional Confucian culture are vital to future social development in China. Emerging empirical research offers empirical support to study the influences of Confucian culture on Chinese society in more detail. Confucian culture has become a hot topic for economic research. However, this paper differs from previous studies on this topic—it has discussed applications of Confucian culture in microeconomics from the perspective of technological innovation. This paper has found that “tradition” may be the opposite of innovation.

American scholar Francis Fukuyama (1995) believes that culture can be divided into high trust culture and low trust culture [105]. High trust culture refers to a society where trust exceeds kinship, while low trust refers to a society where trust only exists in kinship. At the same time, he pointed out that China belongs to low trust culture. In the low trust culture, people believe in people who are related by blood. Therefore, such social relations have also been transplanted to China’s family enterprises, which is specifically manifested in that "outsiders" will not be hired to take on important positions in the enterprise. The long tenure of family business owners may lead to the rigidity of the business and hinder the attempt to completely reform the business. Senior management and the board of directors are mostly composed of business owners with similar experience and similar ideas, which often leads to highly unified thinking and furtherly hinders possible innovation.

Our study, of course, is not without limitations. By examining Confucian culture’s influence on innovation, we focused on the relevant cultural characteristics of an innovation environment that has attracted the attention of prior investigators [106, 107]. However, the impact of culture on innovation efficiency may also include other characteristics, suggesting an opportunity to study a broader spectrum and explore a potentially overarching concept of Confucian culture with the impact of Western modern culture. Moreover, future research should examine whether Confucian cultural characteristics (e.g., the "diversity-orderly structure" of the Chinese Native Soil Society) have varying effects on different phases of the innovation process.

## Supporting information

S1 Raw data (XLS)
